# Validation of the Use of a Smart Band in Recording Spatiotemporal Gait Parameters in the 6-Minute Walk Test

**DOI:** 10.3390/s25082621

**Published:** 2025-04-21

**Authors:** Rosa María Ortiz-Gutiérrez, José Javier López-Marcos, José Luis Maté-Muñoz, Paloma Moreta-de-Esteban, Patricia Martín-Casas

**Affiliations:** 1 Radiology, Rehabilitation and Physiotherapy Department, Nursing, Physiotherapy and Podiatry Faculty, Complutense University of Madrid, Plaza Ramón y Cajal 3, 28040 Madrid, Spain; rosaorti@ucm.es (R.M.O.-G.); jmate03@ucm.es (J.L.M.-M.); pamoreta@ucm.es (P.M.-d.-E.); pmcasas@ucm.es (P.M.-C.); 2 InPhysio Research Group, Health Research Institute of the Hospital Clínico San Carlos (IdISSC), 28040 Madrid, Spain; 3 Comprehensive Integral Rehabilitation, Physiotherapy, Neurorehabilitation Research Group, Complutense University of Madrid, 28040 Madrid, Spain

**Keywords:** gait analysis, wearable electronic devices, digital health, validation study

## Abstract

Wearable monitoring devices, such as smart bands, have emerged as accessible and non-invasive tools for assessing physiological and spatiotemporal gait parameters in various clinical tests. This study aimed to validate the use of the Xiaomi Mi Band 6 for recording gait parameters during the six-minute walk test (6MWT). Seventy participants without gait impairments were recruited, and the measurements obtained with the smart band were compared to reference methods (evaluator, pedometer, and pulse oximeter). The physiological parameter results showed that the smart band demonstrated good accuracy in heart rate monitoring but lower agreement in oxygen saturation measurements. Gait parameters indicated excellent agreement in step count (ICC > 0.9) and step frequency (ICC > 0.75), whereas step length and distance estimations showed higher variability. These findings suggest that the Xiaomi Mi Band 6 is a viable alternative for measuring specific gait parameters, though with limitations in certain aspects of accuracy.

## 1. Introduction

One of the most significant technological advancements of the past five years, driven by the miniaturization of electronic components in mobile phones and the development of sophisticated data management software, has been the emergence of portable monitoring systems or non-invasive biosensors, commonly known as wearables [1]. These devices, which include T-shirts, trainers, belts, watches, and glasses, enable the telemetric recording of health-related parameters, such as biochemical markers in biofluids (sweat, tears, saliva), metabolites, hormones, heart rate (HR), oxygen saturation (OS), stress levels, sleep quality, and calories burned during physical activity [2].

Among these wearables, smart bands have become one of the most popular and widely used technologies in the health sector [3]. These electronic wristbands monitor basic physiological functions and are particularly attractive due to their affordability compared to other smart devices, making them a valuable tool for assessing health status and, more specifically, for conducting functional evaluations. Their user-friendly interfaces, their convenience of integration into daily life, and their capacity to store data and synchronize seamlessly with smart mobile phones make them an ideal option for effortlessly recording health information. These features make smart bands a valuable tool for assessing health status and, more specifically, for conducting functional evaluations [4].

Functional assessment evaluates a person’s performance in specific tasks [5]. In this sense, gait capacity is a multi-level neuromuscular control system, making it an important indicator of functionality. Gait alterations are clinical signs associated with numerous pathologies of the musculoskeletal system, neurological disorders, and aging [6,7,8]. Gait is a key indicator of the impact of pathology on an individual’s health status [9]. Functional gait assessment involves both quantitative and qualitative methods [10]. In clinical practice, the most commonly used systems provide quantitative data on spatiotemporal gait parameters, such as step count, step length, and step frequency, which reflect both timing and distance features of walking such as stride length (SL; the distance between the point of initial contact of one foot and the point of initial contact of the opposite foot), velocity (distance travelled per unit of time), and step frequency (SF) or number of steps (NS) per unit of time [11].

In this context, the 6-min walk test (6MWT) is an established reference tool for functional gait assessment, particularly in relation to cardiopulmonary evaluation [12,13]. The 6MWT was selected due to its wide clinical use, strong reliability, and standardized methodology, making it especially suitable for cardiopulmonary and functional assessment. Clinically, this test is easy to administer, well-tolerated by patients, and has been extensively validated in healthy individuals, as well as in populations with cardiac, pulmonary, neurological, and musculoskeletal conditions, and among geriatric and pediatric patients. It exhibits strong test-retest reliability [14,15,16,17,18,19]. Traditionally, this test has been conducted using analog instruments such as stopwatches, pedometers, and tape measures. In some cases, variables were recorded directly by the assessor. This manual process for preparation, data recording, and information processing is time-consuming, limiting the routine use of this test in clinical practice.

In this regard, smart bands, equipped with geolocation systems and accelerometers, can automatically quantify variables such as distance covered, time spent, NS, and walking speed. Their accessibility, low cost, and ease of use make smart bands particularly promising for use in low-resource clinical settings where traditional gait labs may be unavailable. Additionally, they feature an optical heart sensor that provides data on performance parameters such as HR and OS. Since walking is a submaximal aerobic activity, HR is expected to increase in relation to gait intensity, thus reflecting physiological effort. On the other hand, the monitoring of OS is important for people with effort limitations or pathological conditions. The integration of a smart band into the 6MWT could offer an instrumental system for recording spatiotemporal gait variables together with health-related parameters, addressing the limitations of traditional analog and evaluator systems.

Specifically, the Xiaomi^®^ Mi Band, currently in its sixth version, is one of the most widely marketed smart band models [20]. Regarding its use as a tool for monitoring physical activity, previous studies have demonstrated a good level of precision and agreement of this wristband in step counting and HR monitoring in both adult and geriatric populations, compared to laboratory reference systems [21,22]. Unlike previous studies that evaluated wearable accuracy in daily activity contexts, our study focuses on validating the smart band during a standardized and clinically recognized test (6MWT), which has not been addressed before. Although prior research validated wearables for general activity monitoring, no studies have assessed their performance under standardized clinical protocols like the 6MWT. Validating these devices could provide an accessible, easy-to-use, and cost-effective instrumental system, potentially streamlining the clinical gait assessment process and improving data collection for health sciences research. Therefore, the primary aim of this study was to determine the validity and reliability of a smart band (Xiaomi Mi Band 6^®^, Xiaomi Inc., Beijing, China) as a recording system for spatiotemporal and cardiopulmonary parameters of gait during the 6MWT. This study hypothesized that the Xiaomi Mi Band 6 would provide accurate measurements of HR, OS, and spatiotemporal gait parameters, with moderate variability in other gait parameters, during a standardized 6MWT. Additionally, this study sought to explore the relationship between smart band-derived gait parameters and demographic factors such as gender, age, weight, and height.

## 2. Materials and Methods

### 2.1. Study Design, Setting, and Participants

The study employed a cross-sectional design to evaluate the validity and reliability of a measurement tool, adhering to the guidelines of the Consensus-based Standards for the Selection of Health Status Measurement Instruments (COSMIN). Ethical approval was obtained from the Ethics Committee of the Hospital Clínico San Carlos (internal code: 22/583_P_EC), and the study was conducted in compliance with the principles outlined in the Declaration of Helsinki. All requirements for the protection of personal data were strictly adhered to. The processing, communication, and transfer of personal data complied with the provisions of Organic Law 3/2018, of 5 December, on the Protection of Personal Data and Guarantee of Digital Rights from Spain, as well as Regulation 2016/679 of the European Parliament and of the Council of 27 April 2016, on General Data Protection Regulation.

The study took place at the Faculty of Nursing, Physiotherapy, and Podiatry of the Complutense University of Madrid, where participants were recruited through informational posters distributed in both physical and electronic formats. Eligible participants included individuals of both sexes, aged 18 to 79 years, who were able to walk independently and demonstrated no cognitive impairment (Mini-Mental State Examination score ≥ 24). Participants were excluded if they presented morphological characteristics that interfered with the use of the gait analysis instruments required for the study or had acute or chronic neurological, musculoskeletal, cardiac, or pulmonary conditions that could limit their tolerance to the gait testing procedures. Healthy adults were selected in this first study’s phase to establish baseline reliability before expanding validation to clinical populations.

The sample was recruited using a non-probabilistic, convenience-based purposive sampling strategy, with consecutive cases enrolled until the desired sample size was achieved. Additionally, a “snowball” sampling method was employed, starting with the initial participants to expand recruitment.

### 2.2. Outcome Measures

#### 2.2.1. Six-Minute Walk Test

This Test is designed to measure the maximum distance a person can walk in 6 min. Due to its duration and intensity, it is classified as an aerobic submaximal test, making it a reliable indicator of exercise tolerance [23].

To standardize the administration of this test, the American Thoracic Society (ATS) published clinical practice guidelines in 2002, which include a detailed protocol specifying the required conditions [24]. According to these guidelines, in the present study, the test was conducted along a straight, back-and-forth path on a flat, lightly trafficked hallway surface 25 m in length. The start and end points of the hallway were clearly marked, and additional floor markers were placed every 5 m to facilitate distance measurement

To begin the test, the participant stood behind the starting line, and a verbal cue signaled the initiation of walking, while the stopwatch and step count were activated. At the end of the designated time, the end of the test was indicated with the signal ‘Time, stop where you are’. At no point was it necessary to pause the test, and no participant required the use of walking aids or orthotic devices.

#### 2.2.2. Variables Recorded During Walk Test

To examine the equivalence between data obtained from the smart band and traditional reference methods (stopwatch, pedometer, evaluator-recorded distance and step count, tape measure, pulse oximeter), the variables were recorded as outlined in Table 1.

For HR, measurements were taken as the number of beats per minute. The pulse oximeter relied on spectrophotometry to record this variable, while the smart band used photoplethysmography. Similarly, OS, expressed as the percentage of blood OS, was measured using spectrophotometry with the pulse oximeter and photoplethysmography with the smart band.

Spatiotemporal gait parameters were measured using three distinct methods.

The total NS was recorded using different approaches. Step count was calculated directly by the evaluator. Both the pedometer (Walking style II, OMRON^®^, Kyoto, Japan) and the smart band calculated step count based on changes in acceleration recorded by their integrated accelerometers.

Step length (SL), defined as the distance between the initial contact of one foot and the subsequent initial contact of the opposite foot (expressed in centimeters), was calculated through varying methods. The evaluator used an indirect calculation based on the formula: total steps divided by the total distance travelled (D). Similarly, the pedometer calculated SL using the formula: the NS taken to complete a 10-m distance. The smart band, in contrast, estimates step length using a combination of accelerometer signals and anthropometric data (height and sex) input by the user in the mobile application.

Step frequency (SF), expressed as the NS per minute, was also measured using multiple methods. The evaluator calculated it indirectly with the formula: total steps divided by total time (6 min). The pedometer employed a similar formula, dividing total steps by total time. The smart band provided a direct calculation of SF using its accelerometer.

Lastly, the distance (D), expressed in meters, was determined using distinct techniques for each device. The evaluator calculated it indirectly by counting the number of times the participant completed the length of the hallway (25 m). The smart band measured it directly, while the pedometer used an indirect calculation based on the formula: total steps multiplied by SL.

### 2.3. Study Procedures

All participants were invited to the laboratory via email to participate in the study. After a brief interview to verify inclusion and exclusion criteria, the research team informed participants about the purpose, benefits, and limitations of the study and provided a participant information sheet. Once the information was thoroughly read and understood, participants provided written informed consent. The principal researcher then collected sociodemographic data, including age (calculated in decimal years as the difference between the date of birth and the date of testing), sex (male/female), height (meters), and weight (kilograms). All measurements were conducted in a single 30-min session, with rest periods between tests.

Initially, HR and OS data were recorded. For these measurements, participants remained seated in a quiet environment to ensure optimal conditions for data collection. Upon completion of the test, the same procedure was repeated to obtain post-test measurements. To verify the accuracy of HR and OS measurements, a standard finger pulse oximeter (FS10C ACCARE^®^, Shenzhen, China) was used as the gold standard. HR and OS values from the pulse oximeter were displayed on its digital display, while the corresponding values from the smart band were visible through a mobile phone application (Zepp Life for Android). Outcome variables were recorded simultaneously with the Mi Band 6 (worn on the left wrist) and the pulse oximeter (placed on the index finger of the left hand). During data collection, participants were seated in a relaxed position on a chair. Data were collected from all participants at two different time points: M0, pretest, and M1, immediately after completing the test.

The walking test was conducted along a 25-m corridor, which was clearly marked with cones to delineate the path. Adhesive markers were placed on the floor every 5 m to assist with distance measurement while ensuring they did not interfere with the participant’s natural walking pattern. The distance of the corridor was established by distance measuring laser (Bosch GLM 30, © Bosch Power Tools GmbH 2021, Leinfelden-Echterdingen, Germany).

Prior to performing the 6MWT, body height (in centimeters) and mass (in kilograms) were measured using a precision stadiometer and weighing scale (SECA 769, SECA Corp., Hamburg, Germany) for descriptive purposes. For the calibration of the pedometer, the NS taken to complete a 10-m distance was recorded. Based on these data, a reference SL was established for this device.

Participants wore closed-toe shoes (without heels or platforms) and walked.

Before starting the test, participants were provided with standardized instructions to ensure consistency in test administration. They were informed that the objective was to walk as far as possible within 6 min, following the designated path between the markers. Participants were reminded that they could slow down, stop, or rest if necessary, but should resume walking as soon as they were able. They were explicitly instructed not to run or jog, as the test was designed to assess walking endurance rather than speed. Any questions from participants were addressed before beginning the test to ensure full comprehension of the procedure.

During each test, participants wore a smart band (Xiaomi Mi Band 6) on their left wrist, pre-programmed to record spatiotemporal gait data, and a pedometer. During each test, participants wore a smart band (Xiaomi Mi Band 6) on their left wrist, pre-programmed to record spatiotemporal gait data, and a pedometer securely attached to their waist using a strap with fasteners to minimize its movement.

Simultaneously, a member of the research team accompanied each participant during the tests to measure the time, D, and NS taken. Before and after each test, HR and OS were recorded using both the smart band and a pulse oximeter attached to the participant’s left hand.

All data were recorded on an evaluation sheet and thoroughly reviewed before concluding the assessment session, ensuring any questions from participants were clarified. Additionally, the results of the evaluations were communicated to the participants.

### 2.4. Sample Size

The sample size was determined based on the methodology proposed by Walter et al. and Shieh [25,26] for calculating sample sizes in validation studies using the intraclass correlation coefficient (ICC). A total sample size of 70 subjects was estimated to detect an ICC of 0.8 with a 95% confidence interval ranging from 0.7 to 0.9.

### 2.5. Data Analysis

Statistical analyses were conducted using Jamovi Software 2.3 (2022) for R (REF A-Rosa). A descriptive analysis of all study variables was performed by calculating mean values, standard deviations (SD), and 95% confidence intervals (CI).

Before performing the data analysis, normality and homogeneity of variance were assessed. Normal distribution was evaluated using the Shapiro–Wilk test, while homogeneity was assessed using Levene’s and White’s tests. Descriptive statistics are presented as mean ± SD.

To evaluate the agreement between HR, OS, and gait parameters obtained from the smart band and the reference measures. The intraclass correlation coefficient (ICC) was calculated using a single-measure, two-way mixed-effects model with absolute agreement. ICC values were interpreted as follows: excellent (>0.9), good (0.76–0.9), moderate (0.5–0.75), and poor (<0.50).

Additionally, a Bland–Altman plot analysis was employed to assess agreement between the smart band and standard methods. In the resulting scatterplot, the *Y*-axis represents the differences between paired measurements (A–B), while the *X*-axis shows the average of these measurements [(A + B)/2]. This approach helps to identify systematic bias (i.e., the magnitude and direction of the differences) and to calculate the 95% limits of agreement (LOA) as 1.96 × SD above and below the mean difference [27].

## 3. Results

### 3.1. Participants

The study group comprised 70 participants (45 women and 25 men; mean age 28.8 ± 13.7 years) without gait alterations. No missing data were reported. A detailed description of the participants’ characteristics is presented in Table 2.

### 3.2. Agreement Between Methods Cardiopulmonary Parameters 

**Figure 1:**
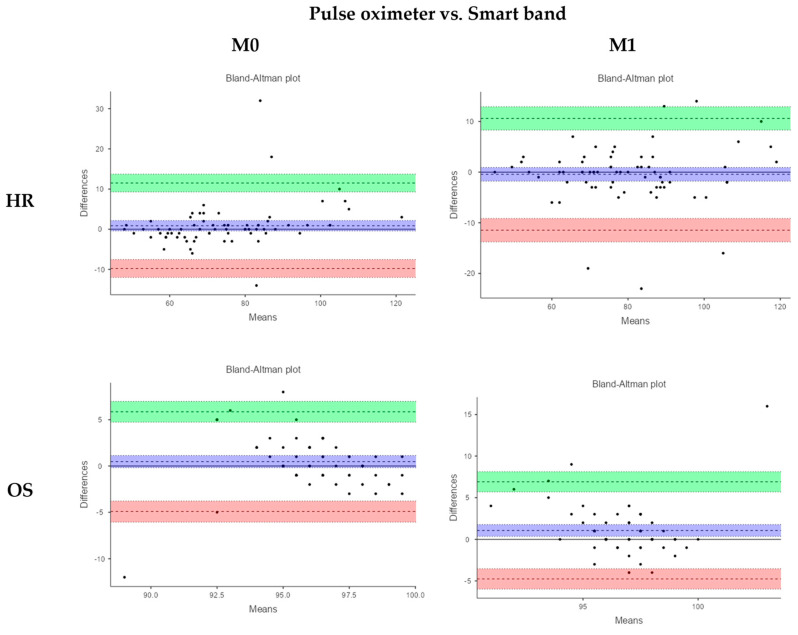
Bland–Altman plots of relative agreement between devices for HR (heart rate) and OS (oxygen saturation).

Regarding physiological variables (Table 3, Figure 1), the ICC analysis indicates excellent agreement between the devices for measuring HR at both pre-test (ICC: 0.94; 95%CI: 0.90 to 0.96) and post-test moments (ICC: 0.95; 95%CI: 0.91 to 0.97), suggesting that both provide comparable results.

The Bland–Altman analysis reveals a low measurement bias for both M0 (MD = 0.9, LoA = −9.8 to 11.5) and M1 (MD = −0.4, LoA = −11.5 to 10.6). However, the wide limits of agreement (±11.5 units) suggest that individual measurements may exhibit relatively large discrepancies.

For OS, the results indicate moderate to low agreement between the devices at both measurement points, with ICC values of 0.31 (95% CI: 0.08 to 0.51) and 0.17 (95% CI: 0.04 to 0.38), respectively. The Bland–Altman analysis demonstrates a low bias at both time points (M0: MD = 0.4, 95% LoA = −4.9 to 5.8; M1: MD = 1.1, 95% LoA = −4.8 to 6.9), with a discrepancy range of ±5–7 units.

It is important to note that these results are based on a sample of healthy individuals. However, these discrepancies could be clinically relevant in specific contexts.

### 3.3. Agreement Among and Between Methods’ Gait Parameters

The NS recorded by the three methods showed minimal differences in the mean values, with standard deviations indicating similar variability across methods. An ICC of 0.92 (95% CI: 0.89 to 0.95) suggests excellent agreement between the assessor, pedometer, and smart band for step counting. Furthermore, the narrow CI, well above 0.75, supports this interpretation, indicating high reliability.

For SL, the mean values were comparable across the three methods. However, the ICC of 0.71 indicates good agreement, albeit with greater variability compared to step counting. The relatively wide confidence interval (95% CI: 0.61 to 0.79) suggests some uncertainty regarding the reliability of the measurements.

SF showed slight differences in the mean values, with the smart band recording somewhat lower values compared to the assessor and pedometer. An ICC of 0.82 indicates good agreement, though the wide CI (0.47 to 0.92) reflects some uncertainty in reliability. The lower bound of the CI suggests that the smart band’s measurement of SF may exhibit greater variability or reduced precision compared to the other methods.

For D, the three methods showed similar average values, although the smart band tended to record slightly lower distances. The ICC of 0.81 indicates good agreement between the methods; however, the relatively wide confidence interval (95% CI: 0.73 to 0.87) suggests moderate variability in the reliability of the measurements.

All these results are presented in Table 4.

For NS, ICC values greater than 0.9 demonstrated excellent agreement between the measurements taken by the smart band and those conducted by the evaluator (ICC: 0.92; 95% CI: 0.88–0.95) as well as the pedometer (ICC: 0.91; 95% CI: 0.85–0.94). These findings suggest a high level of consistency between the methods. Furthermore, the narrow confidence intervals, all above 0.75, reinforce the interpretation of high reliability in the smart band’s step count measurements compared to the other methods.

For SF, the comparisons between the smart band and the evaluator (ICC: 0.77) and the smart band and the pedometer (ICC: 0.76) demonstrated good agreement. However, the wide confidence intervals (Evaluator: 95% CI: 0.02 to 0.92; Pedometer: 95% CI: 0.06 to 0.91) highlight substantial variability in the reliability of the measurements.

Regarding SL, the smart band showed moderate agreement with the evaluator’s measurements (ICC: 0.71; 95% CI: 0.57 to 0.81) and the pedometer (ICC: 0.58; 95% CI: 0.41 to 0.72). While the confidence intervals were relatively narrow, these results indicate some discrepancies between methods, suggesting that SL measurements from the smart band may exhibit greater variability compared to the other methods.

Similarly, the results for D showed good agreement between the smart band and the evaluator, with narrow confidence intervals (ICC: 0.81; 95% CI: 0.71 to 0.88). Specifically, the ICC values for the comparison between the smart band and the pedometer indicate moderate agreement (ICC: 0.70; 95% CI: 0.55 to 0.80), with a wider confidence interval than that observed between the smart band and the evaluator, which affects the consistency of these measurements.

**Figure 2:**
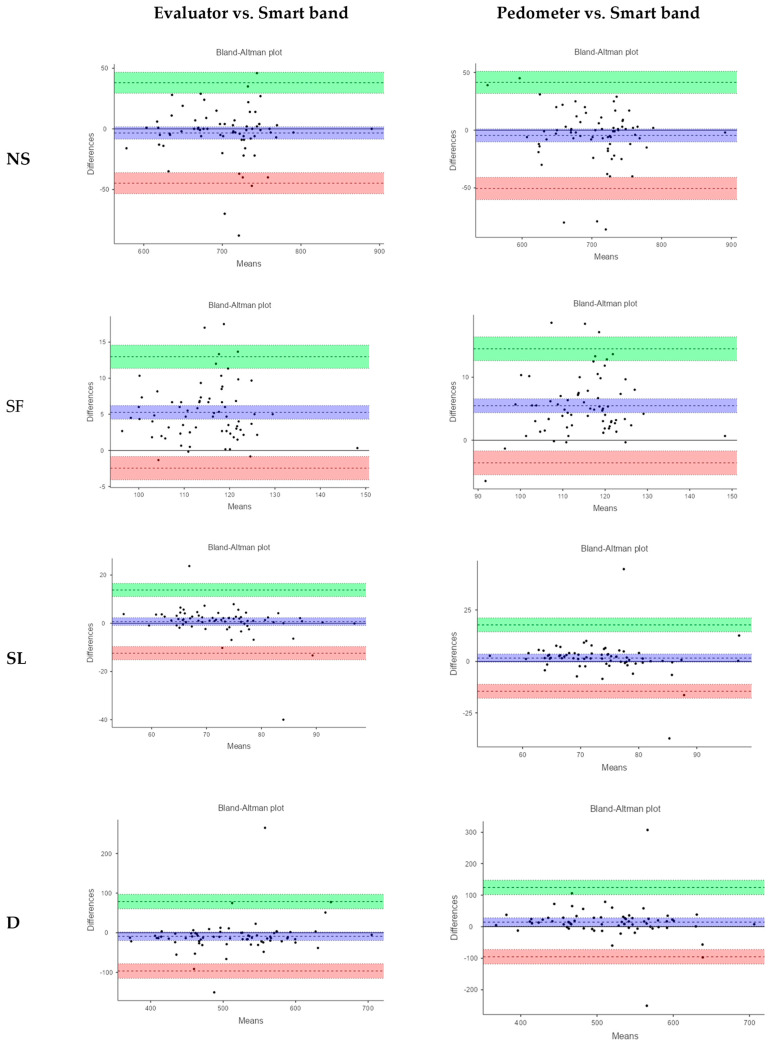
Bland–Altman plots of relative agreement between devices for gait parameters. NS: number of steps; SF: step frequency; SL: step length; D: distance.

Moreover, Bland–Altman plots (Figure 2) reveal that over 90% of the values fall within the limits of agreement across all variables. For step count, the smart band, on average, overestimated by 3.4 steps compared to the evaluator and by 4.5 steps compared to the pedometer, with a variability range of ±40 steps, which was greater in the case of the pedometer.

For SF, the results of Bland–Altman plots suggest a moderate bias in the agreement between the smart band and the reference measurements, with a narrow range of variation (Evaluator: MD 5.3, LoA: −2.5 to 12.9; Pedometer: MD 5.5, LoA: −3.6 to 14.5).

Regarding SL, the mean difference values close to zero indicate low bias, suggesting good agreement between the smart band and the evaluator (MD: 0.7) as well as between the smart band and the pedometer (MD: 1.6). However, the limits of agreement (LoA) were relatively wide for both comparisons (Evaluator, LoA: −12.4 to 13.8; Pedometer, LoA: −14.5 to 17.7).

Finally, for D, the agreement between methods indicates a moderate bias accompanied by a wide range of variability.

These results suggest good agreement between the methods for recording the NS and SF. However, agreement was lower for SL and distance, as evidenced by the wider ranges of the limits of agreement (LoA). Despite these findings, when considering the clinical applicability of the smart band, the observed mean differences across all variables and the broad LoA ranges indicate the presence of both systematic and random biases. The extent of these discrepancies is likely context-dependent.

All results regarding the agreement between methods are summarized in Table 5.

## 4. Discussion

This study aimed to assess the concordance between a popular smart band and conventional methods for recording HR, OS, and spatiotemporal gait parameters during the 6MMT. Smart bands may be useful as a complementary tool for monitoring, though limitations must be considered before clinical application, because they are generally cheaper, lighter, and easier to use than smartwatches and inertial sensors. Specifically, we selected a smart band that is readily available and inexpensive: the Xiaomi Mi Band 6.

Regarding vital signs, we observed good concordance in HR and moderate concordance in OS measurements when comparing data from the smart band to a pulse oximeter, considered the gold standard device. For both variables, there were wide limits of agreement between devices in measurements taken before and after the 6MMT. The lack of accuracy in the measurements could be related to the technology and signal processing algorithms used by each device, as well as the sensor placement for recording these parameters.

In the case of OS, the finger pulse oximeter used in this study consists of two light-emitting diodes (one red and one infrared) and a receiver positioned opposite each other. When light passes through the pulsating bloodstream, hemoglobin tends to absorb infrared light, while unbound hemoglobin absorbs red light. The difference in absorption determines the amount of hemoglobin present in the blood. In contrast, the smart band features a relative OS sensor, which operates in a similar manner, but with the light-emitting diodes and the receiver positioned in parallel. The contact between the sensors and the receiver on the upper wrist is not ideal for detecting the pulse due to the limited penetration of light into the tissues. Moreover, at this location, the sensors are more exposed to hand movements, which can introduce variations in oxygen level readings.

HR recording on the smart band is based on the photoplethysmography (PPG) technique. This technique relies on the emission of green light (LED), which is absorbed by blood vessels during systole and reflected during diastole. Fluctuations in light intensity are recorded by a photodetector, generating a waveform, where the peaks and valleys correspond to HR.

In this sense, the results of a previous study conducted on healthy volunteers showed an excellent concordance correlation coefficient between the Xiaomi Mi Band 5 and an electrocardiogram during a stress test performed on a treadmill. However, these authors indicated that the accuracy of wearable wrist-worn HR monitors varies and depends on the intensity of training [28].

Similarly, a study involving smart bands with similar models (Mi Band 5, Galaxy Fit 2, and PWB-250) in patients with heart disease reported a high level of accuracy for HR measurements during low-intensity exercise and in the recovery phase of a graded exercise test [29].

Regarding gait parameters, to the best of our knowledge, no studies have assessed the validity of using a smart band to record performance in the 6MWT. Our results showed that the NS exhibited the highest degree of agreement among the data collection methods used. This variable is of particular interest since its value is directly obtained. Specifically, it is recorded by counting each step manually (by the evaluator), detecting accelerations via the smart band’s sensors, and using a pedometer. Based on this variable, both the smart band and the pedometer calculate the total D using the formula NS × SL, as well as the SF (NS/time) reported by the evaluator and the pedometer. In this sense, the NS appears to be the most robust spatiotemporal variable, with the least intrasubject variability. Although mean differences were small, the wide limits of agreement observed (e.g., ±40 steps) could still affect clinical decision making, especially in contexts where accurate step counts are essential for monitoring patient progress or tailoring exercise prescriptions.

Regarding the other parameters analyzed, SL was the variable with the lowest concordance among the three methods, although it was moderate. Before performing the test, SL must be manually entered into the pedometer. It is calculated by recording the NS required to travel 10 m, using the formula 10 m ÷ NS. For the smart band, SL is estimated based on height and sex data entered in the mobile app, as well as acceleration recordings. The concordance between the pedometer and the smart band was lower than that observed between the smart band and the evaluator. This variability may be due to reliance on indirect algorithms (e.g., height-based estimations) rather than direct motion tracking, as well as participant diversity in anthropometry and the effect of frequent turns on walking patterns. Additionally, this discrepancy could be explained by the pedometer’s use of a predetermined SL value obtained over a short distance (10 m) prior to the test. In contrast, both the smart band and the evaluator derived SL based on the D covered during the 6MWT.

The distance and SF recorded by the smart band and pedometer were calculated indirectly from NS and SL. In the case of the evaluator, distance was measured by counting the total laps completed on the 25-m circuit, plus the meters travelled in the case of incomplete laps, using a digital meter. The agreement between methods for these variables was good; however, a wide range of agreement values was observed, indicating inter-subject differences.

These differences appear to be related to the inherent variability of the sample in this study. Specifically, distance, SL, and SF are variables influenced by factors such as age, sex, and anthropometric characteristics [30]. The average SL in adult females is estimated at 67 cm, while in males, it is 76.2 cm. Additionally, step frequency is reported as 119 steps per minute in females and 113 steps per minute in males [31]. In the present study, the percentage of female participants was higher (65% women vs. 35% men). Regarding age, this factor has a significant impact on 6MWT performance. Previous research has shown an increase in SL and a decrease in distance with aging [23,32]. To minimize the presence of gait abnormalities and the need for assistive devices, the age range of our sample was set between 18 and 70 years.

Regarding anthropometric characteristics, height is another key factor influencing SL and distance. In our study, the mean height difference between sexes was 14 cm, with the maximum height of women being like the minimum height of men. Therefore, the wide limits of agreement in SL and distance could be explained by the different proportions of female and male participants.

On the other hand, the conditions under which the test was performed also appear to have influenced the recording of these parameters. In our study, the 6MWT was conducted on a 25-m pathway, requiring participants to walk back and forth, which involved frequent 180-degree turns [33]. These turns necessitate gait adjustments, such as shorter steps and a lower step frequency, compared to walking in a straight line. The measurements recorded by the evaluator and the pedometer were not sensitive to these changes. However, the smart band calculates SL and SF based on acceleration data recorded throughout the entire route, including both turns and straight-line walking.

Regarding the clinical implications of this study, the findings suggest that while the Xiaomi Mi Band 6 showed good agreement for heart rate monitoring, its distance estimation demonstrated considerable variability. Therefore, its use for clinical monitoring of gait-related distance should be approached cautiously until further validation is available. Regarding OS recording, although the underestimation by the smart band was relatively small compared to the gold standard, it is important to further investigate the clinical implications of this discrepancy, especially in populations with cardiac and pulmonary conditions.

Among the gait parameters analyzed, the NS recorded by the smart band appears to be the most consistent direct measure, as it is less influenced by intersubject variability and test conditions. Based on this variable, it is possible to indirectly calculate distance, SL, and SF, making them valuable parameters for assessing 6MWT performance.

The results of this validation study will provide evidence-based support for incorporating a smart band into the data collection process for certain spatiotemporal gait parameters, thereby streamlining this process in clinical practice. Additionally, these findings suggest that devices such as the Xiaomi Mi Band 6 hold potential for supporting gait assessment in specific scenarios, such as healthy adults or for monitoring step count, while highlighting the need for further research in clinical populations. Moreover, beyond clinical utility, the technical validation of commercially available wearables contributes to the growing field of low-cost sensor systems for health assessment. Although this study focused on a single device as a first step in validating low-cost wearables in clinical test conditions, future research will include comparisons with other commercial devices both in healthy subjects and in clinical populations.

## 5. Conclusions

The findings of this study suggest that the Xiaomi Mi Band 6 can be a useful complementary tool for gait assessment, particularly for measuring step count and step frequency during the six-minute walk test (6MWT). The high agreement observed in these variables supports its potential for clinical and research use as a low-cost alternative to traditional methods.

However, step length and distance estimations showed considerable variability, likely influenced by participant anthropometry, algorithm-based calculations, and the 25-m test circuit design involving frequent turns. These factors should be considered when interpreting such parameters.

Regarding physiological monitoring, heart rate measurement demonstrated adequate accuracy, while oxygen saturation readings were less reliable, warranting caution in clinical applications where precise values are essential.

Its implementation in functional assessments may improve data collection in routine practice. Further studies should investigate its validity in populations with neuromuscular or cardiopulmonary conditions to assess its broader applicability.

## Figures and Tables

**Table 1:** Variables recorded during walk tests.

Variable Recorded During 6MWT (Unit)	Smart Band	Pedometer	Pulsi Oximeter	Evaluator
HR (beats per minute)	dm		dm	
OS (%)	dm		dm	
NS (steps)	dm	Dm		dm
SL (centimeters)	dm	10-m/NS taken to complete it		total D/total steps
SF (step/second)	dm	total steps/total time		total steps/total time
D (meters)	dm			meters completed

dm: direct measurement; D: distance travelled; HR: Heart Rate; NS: Number of steps; OS: Oxygen saturation; SL: Step length; SF: Step frequency.

**Table 2:** Characteristics of participants.

Variable	$\bar{X}$ ± SD	Female, *n* = 45 (64.3%)		Male, *n* = 25 (35.7%)	
		$\bar{X}$ ± SD	Max–Min	$\bar{X}$ ± SD	Max–Min
Age (years)	28.8 ± 13.7	28.1 ± 14.0	68–18	30.1 ± 13.5	60–18
Height (meters)	1.6 ± 20.1	1.6 ± 0.1	1.7–1.5	1.74 ± 0.1	1.9–1.6
Body mass (kilograms)	65.3 ± 14	59.3 ± 10.2	44.7–86.0	76.2 ± 13.4	60.3–109

$\bar{X}$: mean; SD: standard deviation, *n*: sample; Max: maximum; min: minimum.

**Table 3:** Intraclass correlation coefficient and Bland-Altman analysis between devices.

	M	Device	$\bar{X}$ ± SD	ICC (95% CI)	MD (95% LoA)
HR	M0	Pulse oximeter	75.3 ± 16.6	0.94 (0.90 to 0.96)	0.9 (−9.8 to 11.5)
		Smart band	74.4 ± 15.0		
	M1	Pulse oximeter	79.8 ± 17.0	0.95 (0.91 to 0.97)	−0.4 (−11.5 to 10.6)
		Smart band	80.3 ± 16.7		
OS	M0	Pulse oximeter	96.4 ± 2.4	0.31 (0.08 to 0.51)	0.4 (−4.9 to 5.8)
		Smart band	95.9 ± 2.5		
	M1	Pulse oximeter	97.3 ± 2.2	0.17 (0.04 to 0.38)	1.1 (−4.8 to 6.9)
		Smart band	96.2 ± 2.4		

M: moment; $\bar{X}$: mean; SD: standard deviation; ICC: intraclass correlation coefficient; 95% CI: 95% confidence interval; LoA: limits of agreement; HR: heart rate; OS: oxygen saturation.

**Table 4:** Intraclass correlation coefficient among methods’ gait parameters.

Variable	Method	$\bar{X}$ ± SD	ICC (95% CI)
NS	Evaluator	715 ± 53.9	0.92 (0.89 to 0.95)
	Pedometer	706 ± 56.7	
	Smart band	701 ± 53.2	
SL	Evaluator	73.2 ± 7.6	0.71 (0.61 to 0.79)
	Pedometer	74.52 ± 8.5	
	Smart band	72.5 ± 9.7	
SF	Evaluator	117 ± 8.9	0.82 (0.47 to 0.92)
	Pedometer	118 ± 9.4	
	Smart band	112 ± 8.9	
D	Evaluator	518 ± 68.2	0.81 (0.73 to 0.87)
	Pedometer	523 ± 70.3	
	Smart band	510 ± 76.7	

$\bar{X}$: mean; SD: standard deviation; ICC: intraclass correlation coefficient; 95% CI: 95% confidence interval; NS: number of steps; SF: step frequency; SL: step length; D: distance.

**Table 5:** Intraclass correlation coefficient and Bland–Altman analysis between methods.

Variable	Method		ICC (95% CI)	MD (95% LoA)
NS	Smart band	Evaluator	0.92 (0.88 to 0.95)	3.4 (−38.0 to 44.8)
		Pedometer	0.91 (0.85 to 0.94)	4.5 (−41.5 to 50.5)
SF	Smart band	Evaluator	0.77 (0.02 to 0.92)	5.3 (−2.5 to 12.9)
		Pedometer	0.76 (0.06 to 0.91)	5.5 (−3.6 to 14.5)
SL	Smart band	Evaluator	0.71 (0.57 to 0.81)	0.7 (−12.4 to 13.8)
		Pedometer	0.58 (0.41 to 0.72)	1.6 (−14.5 to 17.7)
D	Smart band	Evaluator	0.81 (0.71 to 0.88)	8.9 (−78.7 to 96.5)
		Pedometer	0.70 (0.55 to 0.80)	14.5 (−95.3 to 124.4)

ICC: intraclass correlation coefficient; 95% CI: 95% confidence interval; MD: mean of difference; LoA: limits of agreement; NS: number of steps; SF: step frequency; SL: step length; D: distance.

## Data Availability

The data associated with the paper are not publicly available but are available from the authors on reasonable request. For additional information, please refer to rosaorti@ucm.es.
